# Prevalence and treatment of patients with eating disorders: Data of a german health insurance

**DOI:** 10.1192/j.eurpsy.2021.324

**Published:** 2021-08-13

**Authors:** K. Herrmann, R. Kaluscha, A. Liebert, J. Von Wietersheim

**Affiliations:** 1 Psychosomatic Medicine And Psychotherapy, Ulm University Hospital, Ulm, Germany; 2 Institute Of Rehabilitation Sciences, Ulm University Hospital, Bad Buchau, Germany

**Keywords:** eating disorders, epidemiology, costs of treatment, health insurance data

## Abstract

**Introduction:**

Few studies have examined the course of eating disorders and the respective treatments based on insurance data, even though they provide representative information.

**Objectives:**

To assess the epidemiology, treatments, duration of illness, costs of treatment in a data set of a public health insurance.

**Methods:**

Data provided by a German health insurance (data from 4.2 million members from 2005-2010). A matched control group based on age and gender without an eating disorder diagnosis was used for comparisons.

**Results:**

2.734 cases with the diagnoses of an eating disorder (anorexia nervosa AN, bulimia nervosa BN or combination ANBN) were identified. More than 92% of the patients were female. The relative risk for personality disorders, depressive disorders, alcohol abuse and obsessive-compulsive disorders was highly increased. Most of the patients with BN (53.04%) or AN (41.57%) were treated in out-patient care, and many were only treated for three months, whereas most of the patients with ANBN were treated for a longer time. 3-19% with BN, AN or ANBN were treated only in in-patient care. The in-patient costs of treatment for the year of the diagnosis were 5471.15€ for BN, 9080.26€ for AN, 10809.16€ for ANBN and 339.37€ for the control group.

**Conclusions:**

Our findings suggest that patients with ANBN diagnosis have a severe and longer course of treatment. Furthermore, contrary to national guidelines for eating disorders, there is a considerable proportion of patients with BN or AN that are treated only in in-patient care.

**Disclosure:**

No significant relationships.

